# Reframing discography through non-binary logic: A dose–response renaissance in spine diagnostics

**DOI:** 10.1016/j.inpm.2026.100750

**Published:** 2026-03-07

**Authors:** Alison Stout

**Affiliations:** Cleveland Clinic, Neurological Institute, Lerner College of Medicine, Cleveland, OH, USA

Discography has been contentious for decades mainly around concerns regarding false-positive responses in asymptomatic subjects and the potential for needle-induced disc degeneration. Critics argued that because discography relies on a subjective pain response, it is inherently vulnerable to psychological confounders, exaggerated pain behaviors, and stimulus-dependent provocation. If excessive pressure can render even normal discs painful, how can the test distinguish pathologic nociception from iatrogenic stimulation?

The first point was settled by a pivotal systematic review by Wolfer, Derby, Lee, and colleagues directly examined the false-positive question [1]. Pooling experimental studies of discography in asymptomatic subjects, they reported a specificity of 0.94 and a false-positive rate of approximately 6% when contemporary standards—particularly pressure limits and concordant pain criteria—were applied. Importantly, they emphasized that discography is a *provocative* test and that stimulus intensity matters. Without controlled pressure thresholds, non-pathologic discs can indeed be rendered painful. With pressure-controlled techniques and defined response criteria, however, the false-positive rate was substantially lower than critics had suggested. The authors concluded that discography’s diagnostic accuracy is highly dependent on technique, thresholds, and disciplined interpretation [1].

Yet diagnostic validity was only half of the debate. A parallel concern emerged from earlier reports suggesting that disc puncture itself might accelerate degeneration or induce chronic pain in previously asymptomatic discs. This possibility cast a long shadow over the procedure’s ethical justification.

Decades later, long-term outcome data now offer important perspective. In a 10-year follow-up study of patients undergoing lumbar total disc replacement, Guyer and colleagues compared discs that had undergone prior discographic injection with those that had not [2]. At more than a decade of follow-up, the rate of subsequent surgery for disc-related pain was not significantly higher in injected discs (10.8%) compared to non-injected discs (8.1%). The only discs at increased risk were those already abnormal or painful on discography—not discs that were simply injected. The authors concluded that discographic injection did not injure normal discs and did not independently increase long-term reoperation risk [2].

Taken together, these bodies of evidence reveal the value and safety of discography performed in a standardized manner. They also show something subtle but crucial: the historical controversy surrounding discography has largely been framed in binary terms. The test is portrayed as either valid or invalid, safe or unsafe, specific or indiscriminate. This framing itself may be part of a deeper issue.

The current publication of *“Quantitative Discometry: Low-Dose Concordant Pain Onset Identifies Sensitized Annular Nociceptors Under Pressure–Volume–Controlled Provocation”* [3] marks an important inflection point in the long-standing debate over provocative discography. For decades, discography has been judged within a binary framework: positive or negative. Derby and Vorobeychik challenge that framing altogether. They reconceptualize it as a controlled dose–response experiment—a quantitative probe of anterior column nociception.

This shift is not semantic. It is logical.

Traditional discography has been evaluated through dichotomy: concordant pain equals “discogenic”; absence of pain equals “not discogenic.” This true/false logic mirrors classical binary systems—1 or 0, positive or negative. The limitations of such reasoning are well known. It compresses complex physiologic behavior into only two possible outcomes and discards gradient information that may be biologically meaningful.

Quantitative discometry replaces this model with a graded mechanical exposure framework. By standardizing fixed-volume increments, capturing static plateau pressures rather than transient peaks, calculating static pressure above opening (ΔP_event), and quantifying cumulative mechanical work (W_event), the procedure becomes a measurable loading experiment. The addition of an energy-equivalent stiffness metric (K_eq) further strengthens its physiologic interpretability.

The study’s core finding is striking. Structurally abnormal discs that reproduced concordant pain did so at relatively low pressure and low cumulative work, yet with moderate-to-severe intensity. In contrast, morphologically normal discs overwhelmingly tolerated high pressure–volume doses without declaring. Notably, many degenerated discs also demonstrated high-dose tolerance despite structural disruption.

Most onset-positive discs declared by approximately 1.7 mL of injected volume, and nearly all by 2.2 mL under slow, pressure-limited infusion. The physiologic interpretation is coherent: low-dose, high-intensity concordant pain most plausibly reflects chemically and mechanically sensitized nociceptors within the annulus and annulus–endplate junction. This is not indiscriminate overdistension; it is threshold sensitization. The low-dose responders appear annular–junctional dominant. Conversely, discs positive at a higher dose may reflect vertebrogenic or alternative mechanisms.

This is non-binary logic applied to clinical science. Instead of asking whether pain is “discogenic” or “vertebrogenic,” the model recognizes that nociceptive territories overlap and that dominance may shift across a spectrum. Degeneration on imaging alone is insufficient to define pain generation; the mechanical–chemical response phenotype becomes the differentiator.

Non-binary logic systems—such as multi-valued logic and fuzzy logic—extend beyond traditional true/false dichotomies. They allow intermediate states, probabilistic membership, and graded thresholds. Truth is not absolute; it may exist along a continuum.

Quantitative discometry embodies this logic. Pain declaration is not merely present or absent; it occurs at a defined dose. Mechanical exposure is not incidental; it is measured. Tolerance is not negative; it is informative. Morphology does not equal causality; it interacts with functional response.

In a binary paradigm, discography attempts to answer: “Is this disc the pain generator?”

In a non-binary paradigm, it asks: “At what mechanical dose does this anterior column structure declare, and what does that reveal about nociceptive sensitization?”

If reproducible, the implications are meaningful.

Discometry may be better understood as a phenotyping tool rather than a binary diagnostic arbiter. Low-dose concordant onset under controlled conditions could identify a sensitized annular–junctional phenotype potentially amenable to biologic or reparative strategies aimed at modulating peripheral sensitization, and suggest endplate pain as less likely.

Future directions include prospective multicenter validation of dose phenotypes, integration with MRI-based endplate and Modic characterization, and correlation with surgical and biologic outcomes. Comparative modeling against basivertebral nerve ablation cohorts will be essential to determine whether low-dose onset and vertebrogenic biomarkers represent overlapping or distinct phenotypic subsets. Ultimately, a unified anterior column pain model will likely require integration of dose–response discometry, imaging biomarkers, and therapeutic response data within a single analytic framework.

The future of spine diagnostics may not be in binary terms. It may depend on embracing the more demanding, but more biologically faithful, logic of gradients, thresholds, and phenotypes. That is the true renaissance this work proposes.

## Declaration of competing interest

The authors declare that they have no known competing financial interests or personal relationships that could have appeared to influence the work reported in this paper.
